# The Role of Nanoparticles in Therapy of Real-World Patients with Pancreatic Cancer: A Scoping Review

**DOI:** 10.3390/cancers17101726

**Published:** 2025-05-21

**Authors:** Ioannis Konstantinidis, Sophia Tsokkou, Dimitrios Katsikeros, Paraskevi Chatzikomnitsa, Menelaos Papakonstantinou, Eftychia Liampou, Evdokia Toutziari, Dimitrios Giakoustidis, Petros Bageas, Vasileios Papadopoulos, Alexandros Giakoustidis, Theodora Papamitsou

**Affiliations:** 1 Department of Medicine, Faculty of Health Sciences, Aristotle University of Thessaloniki, 54124 Thessaloniki, Greece; 2 First Department of Surgery, General Hospital Papageorgiou, Aristotle University of Thessaloniki, 56429 Thessaloniki, Greece; voula.hatzikomnitsa@yahoo.gr (P.C.); menelaospap.md@gmail.com (M.P.); liampouef7@yahoo.gr (E.L.); evdo-t@hotmail.com (E.T.); dgiak@auth.gr (D.G.); pbangeas@gmail.com (P.B.); papadvas@auth.gr (V.P.); algiak@auth.gr (A.G.); 3 Faculty of Medicine, School of Health Sciences, University of Thessaly, 41334 Larissa, Greece; dkatsikeros@uth.gr; 4 Laboratory of Histology-Embryology, Department of Medicine, Faculty of Health Sciences, Aristotle University of Thessaloniki, 54124 Thessaloniki, Greece; thpapami@auth.gr

**Keywords:** pancreatic cancer, pancreatic carcinoma, pancreatic adenocarcinoma, pancreatic neoplasm, nanoparticles, nanomedicine, nanoliposomal irinotecan, nanoparticle albumin-bound paclitaxel, nal-IRI, nab-PTX

## Abstract

**Background:** Pancreatic cancer, one of the most aggressive malignancies, poses significant treatment challenges due to its aggressive nature, late detection, and dense tumor stroma that not only impedes drug delivery but also promotes tumor progression and immune evasion. Nanoparticle-based therapies offer a promising solution by enabling targeted drug delivery, enhancing chemotherapeutic bioavailability, and modulating the tumor microenvironment to reduce systemic toxicity. **Objectives:** This scoping review systematically evaluates real-world clinical evidence to elucidate the translational potential of nanoparticle technologies and identify future directions for integrating these approaches into effective clinical treatment paradigms for pancreatic cancer. **Methods:** A scoping review was conducted following the PRISMA Extension for Scoping Reviews to comprehensively map research on nanoparticle-based therapies in pancreatic cancer over the past 20 years. The research question was identified using the PICOTS framework. A systematic search was performed across PubMed, MEDLINE, Cochrane Library, and Embase on 2 April 2025, using a predefined query: “((pancreatic cancer) OR (pancreatic carcinoma) OR (pancreatic adenocarcinoma) OR (pancreatic neoplasm)) AND (nanoparticles)”. Eligible full-text studies were independently screened by two reviewers, and key study variables were extracted and descriptively synthesized. **Results:** From 2144 initial records, 19 studies were included after screening and eligibility assessment. These studies, published between 2006 and 2023, comprised 14 clinical trials, 2 retrospective cohorts, and 3 case reports, with the majority evaluating nanoparticle albumin-bound paclitaxel, followed by nanoliposomal irinotecan. **Conclusions:** NP-based therapies have emerged as a promising strategy to enhance drug delivery, improve survival outcomes, and reduce toxicity in pancreatic cancer. Clinical evidence, particularly with agents such as nab-PTX and nal-IRI, underscores their potential to overcome the delivery challenges posed by the tumor microenvironment. However, optimizing dosing, biomarker-driven patient selection, and ensuring long-term safety remain critical hurdles. In the coming decade, the integration of nanoparticle therapies into standard-of-care regimens is anticipated to transform pancreatic cancer’s management, contingent upon further rigorous clinical investigations and refined translational strategies.

## Simple Summary

Pancreatic cancer remains one of the deadliest malignancies, largely due to its aggressive biology, late diagnosis, and the presence of a dense tumor stroma that hampers effective drug delivery. Nanoparticle-based therapies offer a promising solution by enabling targeted delivery, enhancing chemotherapy’s bioavailability, and modulating the tumor microenvironment to reduce systemic toxicity. This review evaluated real-world clinical evidence on nanoparticle-based therapies in pancreatic cancer through a scoping review approach guided by the PRISMA Extension for Scoping Reviews. A systematic search was conducted across PubMed, MEDLINE, the Cochrane Library, and Embase on 2 April 2025, which identified 2144 records. Utilizing the PICOTS framework and the pre-set eligibility criteria, studies were independently screened by two reviewers, ultimately including 19 studies published between 2006 and 2023. Clinical evidence indicates that nanoparticle-based therapies, particularly with agents such as nanoparticle albumin-bound paclitaxel and nanoliposomal irinotecan, can improve survival outcomes and reduce toxicity compared with conventional treatments. Nonetheless, challenges remain in dosing optimization, biomarker-driven patient selection, and ensuring long-term safety. Overall, nanoparticle-based approaches show promising potential to be integrated into standard treatment regimens within the coming decade, warranting further clinical investigation to ultimately benefit broader patient populations worldwide.

## 1. Introduction

Pancreatic cancer (PC) is one of the most aggressive and fatal malignancies worldwide, posing a significant global health challenge due to its high mortality rates, late-stage diagnosis, and limited therapeutic efficacy. In the United States, PC ranks as the tenth most common malignancy and the third leading cause of cancer-related mortality, with an estimated 67,440 new diagnoses and approximately 51,980 deaths reported annually as of 2025 [1,2]. In Europe, mortality rates reached 17 per 100,000 among women and 22.3 per 100,000 among men in 2022, indicating a higher prevalence in the male population [3,4]. On a global scale, according to data from the Global Cancer Observatory 2022 (GLOBOCAN), PC was ranked twelfth in incidence, with 510,992 diagnosed cases, and sixth in mortality, with 467,409 recorded deaths [5,6]. The incidence of PC increases with age, with a median age at diagnosis of 71 years based on data from 2018 to 2022, a median age at death of 73 years based on data from 2019 to 2023, and approximately 90% of cases occurring in individuals over 55 years, particularly among those over 70. Moreover, nearly 1.6% of both men and women are diagnosed with PC during their lifetime [1]. Histopathologically, pancreatic ductal adenocarcinoma (PDAC) is the predominant subtype, accounting for over 90% of cases, while additional types such as cystadenocarcinoma and acinar cell carcinoma are also observed [7]. The elevated mortality associated with PC is largely attributable to its asymptomatic early stages, leading to delayed diagnosis and the limited efficacy of current therapeutic interventions [7].

### 1.1. Pancreatic Cancer—Risk Factors

PC is influenced by both modifiable and non-modifiable risk factors. According to the American Cancer Society, tobacco use, obesity, type 2 diabetes (T2DM), chronic pancreatitis, and occupational exposure to carcinogens significantly increase the risk of developing PC. Specifically, smoking is associated with a 25% increased risk, while cessation is linked to a decline in incidence. Additionally, excess body weight, particularly central adiposity and chronic pancreatitis, often consequent to prolonged alcohol consumption, can further increase susceptibility [8,9].

**Figure 1:**
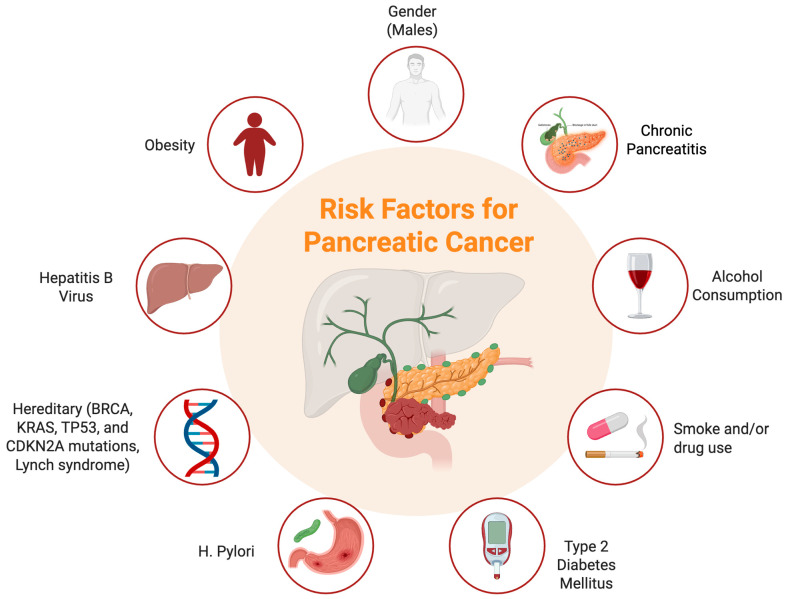
Risk factors for pancreatic cancer development.

Non-modifiable risk factors include advanced age, male gender, and race, with African Americans being more predisposed than Caucasians. Familial predisposition also demonstrates a key role; individuals with a family history of PC or those with inherited genetic syndromes, such as mutations in BRCA, KRAS, TP53, or CDKN2A, as well as Lynch syndrome and hereditary pancreatitis, face a higher risk. Moreover, emerging studies suggest that dietary factors, physical inactivity, certain infections (including *Helicobacter pylori* and hepatitis B virus), heavy alcohol consumption, and gestational diabetes mellitus may contribute to PC risk, although further research is necessary to substantiate these associations (Figure 1) [8,9].

### 1.2. Molecular and Cellular Alterations–Pancreatic Ductal Adenocarcinoma

**Figure 2:**
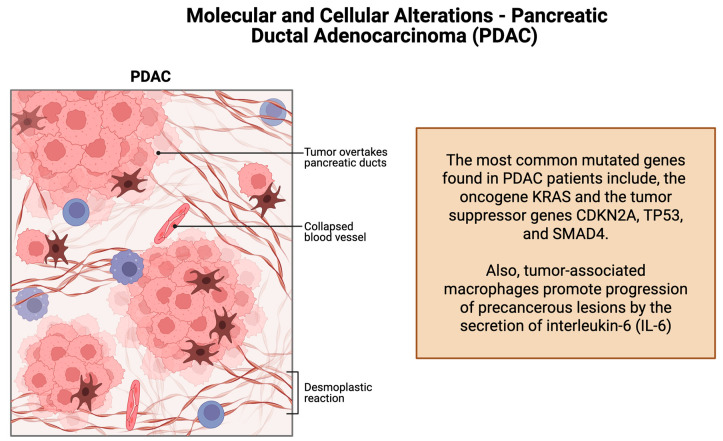
Molecular and cellular alterations—pancreatic ductal adenocarcinoma.

PDAC is predominantly driven by somatic mutations in both oncogenes and tumor suppressor genes. In particular, activating mutations in the oncogene *KRAS* and loss-of-function mutations in the tumor suppressor genes *CDKN2A*, *TP53*, and *SMAD4* are among the most frequently observed genetic alterations in *PDAC* patients (Figure 2) [10]. Moreover, *PDACs* exhibit significant alterations in the composition of immune cell subpopulations, contributing to an immunosuppressive or “cold” tumor microenvironment. Notably, there is an enrichment of regulatory T cells and myeloid-derived suppressor cells, which impede the effective immune-mediated targeting of cancer cells [10]. Also, tumor-associated macrophages promote the progression of precancerous lesions by the secretion of interleukin-6 (IL-6). Immune alterations in *PDAC* negatively impact disease progression by contributing to the lack of responsiveness to immunotherapeutic strategies, including checkpoint inhibition. Elucidating the molecular signals that drive the formation of this immunosuppressive microenvironment may uncover novel therapeutic targets to enhance treatment efficacy [10].

### 1.3. Pancreatic Cancer Therapy

The aggressive nature of PC and its frequent late-stage diagnosis make it one of the most challenging malignancies to treat. Current treatment strategies depend on the patient’s health status, tumor resectability, and the stage of the disease. For tumors confined to the pancreas, without vascular invasion, the Whipple procedure (pancreaticoduodenectomy) is the standard approach, with surgical resection representing the only curative option [11,12,13]. However, only approximately 20% of patients are eligible for surgery at the time of diagnosis. Neoadjuvant therapy, which encompasses both chemotherapy and radiation, is increasingly employed to reduce the tumor burden and improve surgical outcomes in cases of borderline resectability [11,12,13].

Chemotherapy is the first-line treatment for locally advanced and metastatic pancreatic cancer. The FOLFIRINOX regimen, comprising fluorouracil, leucovorin, irinotecan, and oxaliplatin, has demonstrated superior efficacy compared to gemcitabine monotherapy, resulting in a significant increase in survival rates. For patients unable to tolerate FOLFIRINOX, gemcitabine combined with nab-paclitaxel serves as a more tolerable and widely utilized alternative [11,12,13]. Furthermore, emerging personalized treatment approaches include targeted therapies, such as PARP inhibitors (e.g., olaparib) for patients with BRCA mutations, as well as tyrosine kinase inhibitors. Additionally, immunotherapy—which includes immune checkpoint inhibitors—has demonstrated potential in other malignancies; however, its application in pancreatic cancer remains under investigation due to the immunosuppressive tumor microenvironment [11,12,13]. In advanced cases, radiation therapy is frequently employed in conjunction with chemotherapy to alleviate symptoms and achieve local tumor control [11,12,13].

Recently, the U.S. Food and Drug Administration (FDA) approved NALIRIFOX, a novel chemotherapy regimen that integrates liposomal irinotecan (nal-IRI), fluorouracil, leucovorin, and oxaliplatin [11,12,13]. Preliminary evidence indicates that NALIRIFOX exhibits enhanced efficacy in the treatment of metastatic pancreatic cancer. This advancement reflects ongoing efforts to improve patient outcomes and treatment efficacy. Concurrently, current research is focusing on biomarker-driven therapies, modulation of the tumor microenvironment, and innovative drug delivery systems [11,12,13].

### 1.4. Nanoparticles in Pancreatic Cancer Therapy

Nanoparticles (NPs) have revolutionized cancer treatment by offering innovative solutions to overcome the limitations of conventional therapies. These nanoscale particles, ranging from 1 to 100 nanometers in size, are meticulously engineered to enhance drug delivery, optimize therapeutic efficacy, and minimize systemic toxicity. NPs can be selectively designed to target only tumor cells by exploiting the enhanced permeability and retention (EPR) effect, or through active targeting approaches using ligands that bind to cancer-specific biomarkers. This targeted delivery facilitates the accumulation of therapeutic agents at the tumor site while reducing off-target effects, thereby potentially improving clinical outcomes [14,15,16,17,18,19,20,21,22,23,24,25,26].

Various types of NPs, including liposomes, polymeric NPs, metallic NPs and lipid-based NPs (LBNPs), as well as novel mRNA-lipoplex vaccines targeting somatic mutation-derived neoantigens, have been implemented in cancer therapy. These nanoscale carriers can encapsulate chemotherapeutic agents, thus facilitating controlled and programmed release at the target site while enhancing drug stability in systemic circulation and minimizing off-target complications [9,10,11,12,13,14,15,16,17,18,19,20]. Additionally, multifunctional NPs are engineered to combine therapeutic and diagnostic capabilities, thereby enabling real-time imaging and treatment monitoring. NPs also play a crucial role in overcoming drug resistance by delivering combination therapies or modulating the tumor microenvironment. For instance, lipid–polymeric hybrid NPs have demonstrated enhanced radiosensitivity and radiation-triggered drug delivery, providing synergistic effects in cancer treatment. Despite this promise, challenges such as scalability, biocompatibility, and regulatory approval continue to represent critical areas of ongoing research [16,17,18,19,20,21,22,23,24,25,26,27].

PC remains one of the most formidable challenges in oncology due to its aggressive biology, late-stage diagnosis, and intrinsic resistance to conventional therapies such as chemotherapy and radiotherapy. The complex and dense tumor stroma, which is characteristic of PC, not only serves as a physical barrier that hinders the effective penetration of therapeutic agents but also fosters a microenvironment that promotes tumor growth and immune evasion. NPs have emerged as a promising means to overcome these impediments, owing to their ability to enhance targeted drug delivery, improve the bioavailability of chemotherapeutic agents, and modulate both the tumor microenvironment and immune responses. These engineered nanoplatforms enable the precise delivery of drugs directly to tumor sites, thereby potentially increasing therapeutic efficacy while minimizing systemic toxicity. Given these innovative capabilities, the present scoping review aims to systematically explore and critically appraise the application of NP-based therapies in the management of PC, with a specific focus on evidence derived from real-world clinical settings. By synthesizing the current research on NP-mediated drug delivery systems, stromal barrier modulation, and immune microenvironment alteration, this review seeks to elucidate the translational potential of NP technologies and identify future directions for integrating these approaches into effective clinical treatment paradigms for PC.

## 2. Materials and Methods

A scoping review approach was adopted for this project in lieu of a formal systematic review, due to the diverse approaches encountered in studies addressing NP applications in PC therapy. This methodological choice was made to comprehensively map the extent of the research, including variability in study designs, implementation strategies, and outcome measures, rather than attempting to synthesize data from highly disparate sources into a single analysis. By using a scoping review framework, we aimed to delineate critical gaps in the current literature, particularly regarding the effectiveness of novel NP strategies in overcoming the unique challenges of PC in real-world clinical settings, and identify key advancements. This approach not only facilitates the characterization of existing evidence on therapeutic effectiveness, it also provides a foundational understanding of implementation strategies across diverse patient populations and elucidates the practical challenges associated with translating NP technologies from experimental models to routine clinical practice, thereby informing future systematic reviews and guiding translational research efforts in this evolving field. A detailed protocol for this scoping review was systematically developed, and all results were documented following the Preferred Reporting Items for Systematic Reviews and Meta-Analyses (PRISMA) checklist adapted for scoping reviews, as detailed in Supplementary Table S1.

### 2.1. Identifying Research Questions

To clarify the research query, a PICOTS (Population, Intervention, Comparator, Outcome, Time, Study design) framework was employed, as detailed in Table 1. Thus, the following review question was developed: “Among adult (≥18 years) patients with PC, what is the evidence regarding the efficacy of NP-based pharmacological approaches in improving overall survival (OS), progression-free disease (PFS), disease control rate (DCR), partial remission (PR), complete remission (CR), stable disease (SD) and treatment-emergent adverse events (TEAEs), compared with conventional pharmacological regimens over the past twenty years?”.

### 2.2. Identifying Relevant Studies

A comprehensive search was conducted on 2 April 2025, across multiple biomedical databases, including PubMed, MEDLINE, Cochrane Library, and Embase, using the following query string: “((pancreatic cancer) OR (pancreatic carcinoma) OR (pancreatic adenocarcinoma) OR (pancreatic neoplasm)) AND (nanoparticles)”. References were reviewed for the identification of any additional relevant articles.

### 2.3. Study Selection—Eligibility and Screening

The review was restricted to full-text research studies published in peer-reviewed journals in the English language. Eligible studies had to meet the following criteria: the study must include adult (≥18 years) patients with PC of any histopathological type or clinical stage; the study design must be a clinical trial, prospective or retrospective cohort, or case report; the study should focus primarily on the effectiveness of NP-based regimens for treatment of PC and, when available, a direct comparison with conventional pharmacotherapy; the study must have been published during the past twenty years, i.e., between 2005 and 2025. Duplicate records, review articles, systematic reviews and meta-analyses, protocols and guidelines, animal studies, conference abstracts and presentations, preprints, clinical trials under patient recruitment or without published results, ongoing clinical trials, and studies deemed irrelevant were excluded.

After the exclusion of articles based on the above criteria by both the automated tools and the researchers, the final set of articles was retrieved. To ensure accuracy and objectivity, two independent reviewers (I.K. and S.T.) initially screened the titles and abstracts in a double-blinded process. For studies that passed this initial screening, the full texts were obtained and further evaluated to determine their final eligibility. Any discrepancies during the screening process were resolved by a third reviewer (T.P.).

### 2.4. Data Charting

Key variables were systematically extracted from all included studies by the primary researchers (I.K. and S.T.), comprising the first author, publication year, study design, number of participants, NP-based regimen discussed, pharmacological regimens assessed, mean age of participants in years, median overall survival in months, median progression-free disease in months, disease control rates, partial remission rate, complete remission rate, stable disease rate, treatment-emergent adverse events reported, and finally, the key findings as a conclusion.

### 2.5. Collating, Summarizing, and Reporting Results

The extracted data were synthesized into results tables. Given the scoping nature of this review, a meta-analysis was not conducted; instead, a descriptive synthesis of the principal findings was performed to explore and evaluate the application of NP-based therapies in the management of PC, particularly within real-world clinical settings.

## 3. Results

**Figure 3:**
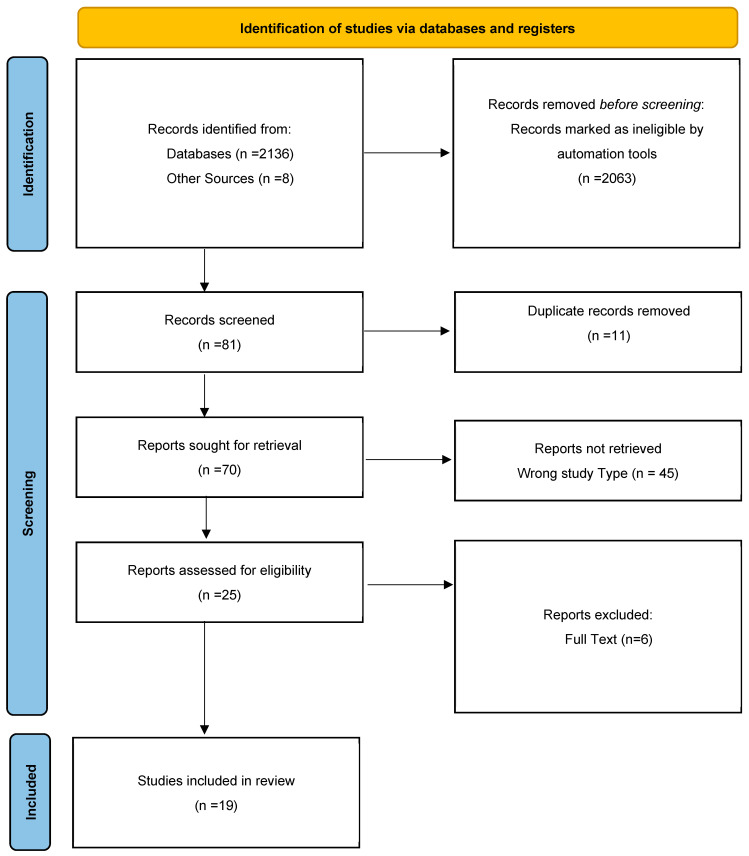
PRISMA flow diagram.

The PRISMA flow diagram (Figure 3) outlines the review selection and exclusion process. Initially, a total of 2144 records were retrieved from the aforementioned databases. Automated screening excluded 2063 records, leaving 81 records for further consideration. Of these, 11 duplicate records were removed manually. Subsequently, 45 studies were excluded based on ineligible study design, as determined through title and abstract screening. After a full-text review of the remaining 25 articles, a total of 19 studies met the inclusion criteria and, thus, were included in the review.

The included studies were published between 2006 and 2023 (Table 2 and Table 3). Most of the studies (n = 14) were clinical trials, whereas two were retrospective cohorts and three were case reports. Most of the studies (n = 10) assessed nanoparticle albumin-bound paclitaxel, whereas four evaluated nanoliposomal irinotecan, two weighed both of the aforementioned regimens, one considered CYT-6091, one NK105, and finally, one reviewed nanoliposomal cisplatin.

## 4. Discussion

Pancreatic cancer, most commonly presenting as PDAC, remains one of the most dreadful malignancies due to its limited therapeutic options and dismal survival outcomes. The integration of NP-based technologies into PC treatment has yielded significant advancements, notably in enhancing drug delivery, improving therapeutic efficacy, and reducing systemic adverse effects and toxicities. By modifying pharmacokinetics, increasing tumor penetration, and refining second-line treatment strategies, nanomedicine has fundamentally transformed treatment paradigms for PC.

### 4.1. Nanoparticle Albumin-Bound Paclitaxel (nab-PTX) in Pancreatic Cancer

**Figure 4:**
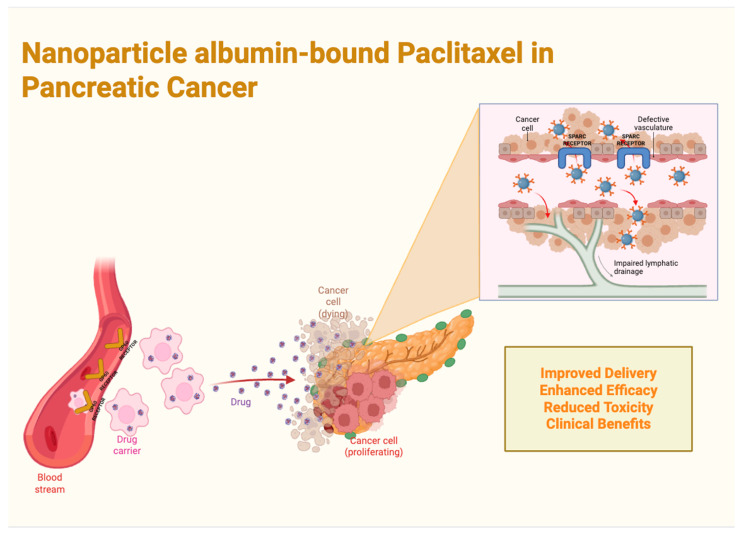
Nanoparticle albumin-bound paclitaxel in pancreatic cancer: mechanisms of action and advantages compared to conventional chemotherapy regimens.

Nanoparticle albumin-bound paclitaxel (nab-PTX) accounts for a major advancement in PC treatment by enhancing drug delivery and tumor penetration while diminishing treatment-emergent adverse events (TEAEs) and toxicities correlated with conventional paclitaxel. This formulation leverages albumin as a carrier to accelerate active transport through gp60 receptors and the caveolin-1-SPARC pathway, permitting more efficient intratumoral drug accumulation [40]. This mechanism of action results in significant clinical benefits, comprising improved OS and PFS, superior tumor response rates, and enhanced chemotherapy delivery to neoplastic tissues (Figure 4) [40].

Regarding the mechanism of action and tumor microenvironment modulation, nab-PTX demonstrates a dual function as both a cytotoxic agent and a tumor stroma modifier. In a phase I/II clinical trial by Von Hoff DD et al. in 2011, critical preclinical evidence demonstrated that nab-PTX depletes the tumor stroma, thereby enhancing intratumoral gemcitabine concentrations through increased vascular perfusion [40]. This stromal depletion is significant, as the dense fibrotic stroma characteristic of pancreatic tumors frequently inhibits effective drug delivery and, therefore, nab-PTX facilitates chemotherapy penetration, improving drug accumulation and enhancing tumor cell death [40]. Additionally, the study demonstrated that elevated expression of Secreted Protein Acidic and Rich in Cysteine (SPARC) in the tumor stroma is associated with improved survival rates, supporting the hypothesis that targeting stromal components is essential for maximizing treatment efficacy. These findings underline the significance of integrating stromal-depleting therapies with standard chemotherapy regimens to optimize drug penetration and overall therapeutic outcomes [40]. Furthermore, an alternative nab-PTX formulation—NK105, a polymeric micelle formulation of paclitaxel—was evaluated by Hamaguchi T et al. in 2007 for its pharmacokinetic advantages. NK105 exhibited a plasma area under the curve (AUC) 15 to 20 times higher than that of conventional paclitaxel, thereby augmenting the drug stability and improving tumor targeting [42]. The study further demonstrated that lower tissue distribution of NK105 resulted in reduced toxicity in normal tissues, particularly neurotoxicity, suggesting that it may offer an alternative paclitaxel delivery system with a more favorable adverse event profile [42].

Concerning clinical efficacy and survival benefits, the pivotal MPACT randomized control trial by Von Hoff DD et al. (2013) verified that nab-PTX in combination with gemcitabine significantly enhanced survival outcomes compared with gemcitabine monotherapy in patients with mPDAC who had not received prior chemotherapy [38]. The mOS improved by 1.8–8.5 (95% CI 7.89–9.53) months versus 6.7 (95% CI 6.01–7.23) months in the gemcitabine-only arm, with long-term survival rates similarly favoring the combination therapy (35% vs. 22% at one year and 9% vs. 4% at two years). Similarly, the mPFS increased by 31%, with values of 5.5 (95% CI 4.5–5.9) months in the control arm compared to 3.7 (95% CI 3.6–4.0) months in the control group, thereby reducing the risk of disease progression or death [38]. Moreover, the study indicated that declining serum CA19–9 levels were strongly correlated with improved survival, reaffirming the prognostic significance of this biomarker, and that nab-PTX was shown to enhance chemotherapy delivery by modulating the tumor stroma to facilitate increased drug penetration [38]. Furthermore, a subsequent phase II randomized trial by Macarulla T et al. (2019) evaluated nab-PTX in patients with locally advanced or advanced PDAC and an Eastern Cooperative Oncology Group (ECOG) performance status of 2. The findings confirmed that the regimen was both efficacious and tolerable in this more vulnerable patient population, with mOS and mPFS sustained at 8.5 and 5.5 months, respectively, values comparable to the results of the MPACT trial [37]. However, baseline patient heterogeneity within the study cohort may have influenced tolerability and outcomes, underscoring the need for more robust patient stratification tools. Moreover, the incorporation of objective biomarkers, such as albumin levels, inflammatory markers, and body composition, could aid in refining patient selection and optimizing treatment strategies [37].

In this context, Hasegawa et al. (2019) conducted a clinical trial in patients with unresectable advanced pancreatic adenocarcinoma or adenosquamous carcinoma aged over 75 years. Despite lower-than-expected chemotherapy completion rates, the combination of nab-PTX and gemcitabine improved mOS and mPFS in elderly patients compared to gemcitabine monotherapy, as confirmed by an mOS of 10.3 (95% CI 8.2–12.5) months and an mPFS of 7.0 (95% CI 6.0–8.1) months, with favorable disease control observed even in stage IV cases [36]. While this regimen remains feasible with appropriate dose management, elderly patients, particularly those with sarcopenia, appear more susceptible to non-hemotoxic adverse events, highlighting the need for further large-scale studies and improved patient selection criteria [36]. Furthermore, a phase II clinical trial by Hosein PJ et al. (2013) investigated nab-PTX monotherapy in patients with unresectable locally advanced or metastatic PC who had progressed on gemcitabine-based therapy, reporting an mOS of 7.3 (95% CI 2.8–15.8) months and an mPFS of 1.7 (95% CI 1.5–3.5) months, with 37% of patients surviving beyond thirteen weeks. Notably, one patient remained on nab-PTX therapy for nearly two years, establishing the feasibility of prolonged administration of nab-PTX in selected individuals [39].

In the context of peritoneal metastases associated with advanced PC, patients confront unique therapeutic challenges due to inadequate drug penetration into the tumor core. In this setting, pressurized intraperitoneal aerosol chemotherapy (PIPAC) has emerged as an innovative drug delivery technique to overcome these impediments. In a phase I clinical trial, Ceelen W et al. (2022) evaluated aerosolized nab-PTX and established that the regimen was well tolerated while achieving enhanced tumor uptake over multiple treatment cycles. Importantly, systemic exposure remained significantly lower compared to intravenous administration, thereby diminishing the risk of adverse events [33]. The study reported a histological response rate of 35% among patients receiving two or more PIPAC cycles, with the survival data indicating that more than half of the patients survived beyond one year. These promising findings warrant further clinical investigation, particularly to refine dose selection and sequencing strategies for this modality [33].

In a phase II randomized clinical trial by Sohal DPS et al. (2021), nab-PTX was evaluated in the neoadjuvant setting as part of a perioperative chemotherapy regimen for treatment-naïve pancreatic adenocarcinoma without metastases. The study reported a slightly higher mOS in the nab-PTX plus gemcitabine cohort, at 23.6 (95% CI 17.8–31.7) months, compared to 23.2 (95% CI 17.6–45.9) months in the modified FOLFIRINOX arm. However, this difference did not represent an improvement in OS when compared with historical perioperative chemotherapy trials [34]. Additionally, while a modestly higher incidence of neutropenia was observed in the nab-PTX cohort (27% versus 19%), severe diarrhea was more common in the mFOLFIRINOX arm (11% versus 4%), although neither of these differences was statistically significant [34].

With reference to toxicity and other safety considerations, nab-PTX necessitates meticulous management, particularly concerning neurotoxicity and myelosuppression. The MPACT trial by Von Hoff DD et al., in 2013, identified peripheral neuropathy as a noteworthy adverse effect, although these symptoms were reversible with treatment suspension and dose modifications [38]. Additionally, sepsis and pneumonitis risks were effectively managed through protocol adjustments, and myelosuppression was manageable with appropriate supportive care [38]. Comparative safety analyses by Hosein PJ et al. (2013) established improved tolerability with nab-PTX monotherapy, characterized by minimal non-hematological toxicities and an absence of Grade 3–4 peripheral neuropathy [39]. Moreover, Macarulla T et al. (2019) noted that a lower dose maintained survival benefits while reducing toxicity and enhancing tolerability, particularly among more fragile patients with an ECOG performance status of 2 [37]. Similarly, Otsubo M et al. (2021) reported taxane-related cystoid macular edema (CME) as a rare yet noteworthy adverse event affecting bilateral central vision in a patient with stage IV PC undergoing a nab-PTX plus gemcitabine regimen [45]. Encouragingly, CME resolved after the suspension of nab-PTX and treatment with local dorzolamide, suggesting a potential management strategy for similar cases [45].

As treatment regimens for mPDAC continue to advance, integrating nab-PTX with emerging therapies demonstrates considerable promise. For instance, Azmi AS et al., in 2020, assessed the combination of nab-PTX with selinexor, a nuclear export inhibitor targeting XPO1. By retaining tumor suppressor proteins within the nucleus of the tumor cells, selinexor enhances cancer cell apoptosis and suppresses PDAC proliferation [35]. Preclinical evidence from this study established that the combination was particularly effective in PDAC stem cells and patient-derived xenograft models, indicating that targeting multiple cancer cell survival pathways could overcome chemotherapy resistance mechanisms and further improve treatment outcomes [35]. Additionally, Huang X et al. (2021) reported a case of a patient with stage IV primary squamous-cell carcinoma of the pancreas harboring a deleterious BRCA2 somatic mutation. The combination of cisplatin and nab-PTX in this patient resulted in significant tumor reduction and prolonged survival, along with improved tumor resectability, and represented the longest reported survival for metastatic pancreatic squamous-cell carcinoma to date. These findings underscore the potential clinical impact of such combination therapies [44].

### 4.2. Nanoliposomal Irinotecan (nal-IRI) in Pancreatic Cancer

**Figure 5:**
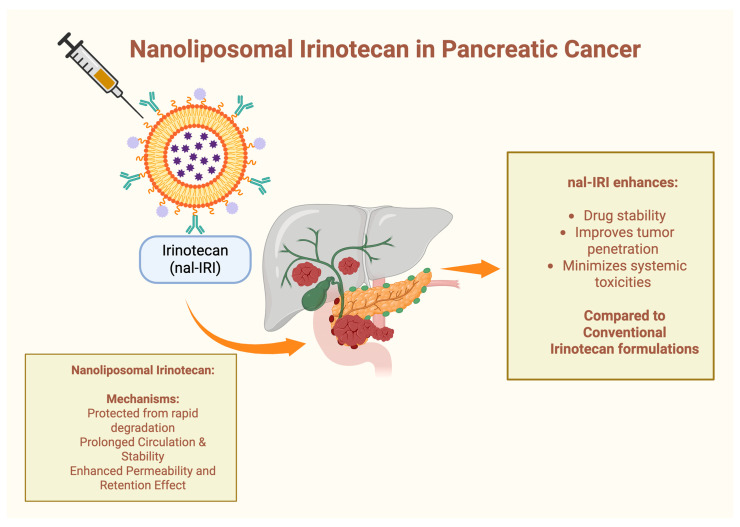
Nanoliposomal irinotecan in pancreatic cancer: mechanism of action and advantages compared to conventional irinotecan-based regimens.

Nanoliposomal irinotecan (nal-IRI) represents a significant advancement in the treatment of mPDAC, markedly altering the disease’s prognosis. By encapsulating irinotecan within liposomes, nal-IRI enhances drug stability, improves tumor penetration and minimizes systemic toxicities compared to conventional irinotecan formulations (Figure 5). Multiple clinical trials have demonstrated its efficacy, establishing nal-IRI as an essential component of second-line treatment regimens following gemcitabine-based therapies.

With respect to the clinical efficacy and survival benefits of nal-IRI, the NAPOLI-1 trial by Wang-Gillam A et al. (2016) demonstrated that the combination of nal-IRI with 5-fluorouracil (5-FU) and folinic acid/leucovorin (LV) is superior to fluorouracil monotherapy for patients with metastatic PC previously treated with gemcitabine-based regimens [32]. Specifically, the trial reported a significant improvement in mOS and mPFS to 6.1 (95% CI 4.8–8.9) and 3.1 (95% CI 2.7–4.2) months, respectively, compared to 4.2 (95% CI 3.3–5.3) and 1.5 (95% CI 1.4–1.8) months in the monotherapy cohort, respectively. In addition to these survival benefits, the combination regimen enhanced objective tumor response rates and significantly reduced CA19–9 levels, thereby reaffirming its viability as a treatment option [32]. Further evidence supporting the value of nal-IRI in second-line treatment came from a retrospective cohort study by Park SJ et al. in 2021, which evaluated nal-IRI plus 5-FU/LV in patients with progressed or recurrent mPDAC previously treated with gemcitabine-based therapy. This study reported survival outcomes consistent with the NAPOLI-1 trial, with an mOS of 7.0 (95% CI 6.0–7.9) months and an mPFS of 2.8 (95% CI 1.8–3.7) months, along with manageable toxicities [30]. However, a higher incidence of Grade 3–4 neutropenia was noted compared to the NAPOLI-1 findings, emphasizing the necessity for vigilant hematological monitoring. Notably, nal-IRI remains a first-line treatment in only 20–40% of patients with metastatic PC, underscoring the importance of patient selection and individualized dosing strategies [30].

Another retrospective study by Glassman DC et al. (2018) further elucidated the role of nal-IRI in sequencing treatment strategies. Patients who received nab-PTX with gemcitabine, followed by nal-IRI plus 5-FU/LV as a second-line therapy, without prior irinotecan exposure, achieved an mOS of 9.0 months and an mPFS of 4.8 months. In contrast, patients who had undergone prior treatment with the FOLFIRINOX regimen (and consequently received nal-IRI as a third-line treatment) exhibited an mOS of 4.1 months and an mPFS of 2.2 months. These findings advocate for the strategic integration of nal-IRI into the treatment algorithm to prolong survival [31]. Complementing these data, a case report by Assi HA et al. (2020) described a rare instance of long-term survival in a patient with mPDAC under nal-IRI + 5-FU/LV therapy following disease progression on a nab-PTX plus gemcitabine regimen [46]. The patient exhibited a sustained response for over two years, receiving 58 cycles without dose adjustments. Remarkably, the reported PFS and OS were 31 and 40 months, respectively, underscoring the potential benefits of this regimen and the need for further refinement of sequential regimen therapies [46]. Further evidence was derived from the NAPOLI-3 phase III randomized clinical trial by Wainberg ZA et al. (2023), which compared a quadruplet regimen of NALIRIFOX (incorporating nal-IRI) with FOLFIRINOX, as evaluated in the PRODIGE 4/ACCORD 11 phase III clinical trial by Conroy T et al. (2011). In NAPOLI-3, the mPFS was 7.4 (95% CI 6.0–7.7) months for NALIRIFOX, compared to 6.4 (95% CI 5.5–7.2) months for FOLFIRINOX in PRODIGE, insinuating that nal-IRI confers advantages over conventional irinotecan-based regimens [28]. Importantly, nal-IRI was correlated with a lower incidence of neutropenia and neuropathy relative to FOLFIRINOX, positioning it as a more tolerable alternative for patients with compromised performance status and those at risk of TEAEs [28,30,47]. Moreover, in the context of sequential combination chemotherapy regimens, Bockorny B et al. (2021) conducted a phase IIa clinical trial (COMBAT/KEYNOTE-202) investigating the incorporation of motixafortide to potentiate immune responses by increasing circulating lymphocytes and neutrophils, in combination with pembrolizumab and nal-IRI plus 5-FU/LV, in patients with de novo mPDAC who had documented radiographic progression after first-line gemcitabine-based therapy. This regimen demonstrated clinical benefit, with durable responses, reflected in an mOS of 6.6 (95% CI 4.5–8.7) months, an mPFS of 3.8 (95% CI 1.6–5.1), and a notably lower rate of severe neutropenia (7%, compared to 27% in the NAPOLI-1 trial), likely attributable to CXCR4 inhibition by motixafortide [29].

Regarding toxicity and safety considerations, despite its established efficacy, nal-IRI is correlated with specific toxicity concerns, predominantly hematological toxicity. In the NAPOLI-1 trial, higher rates of Grade 3–4 neutropenia were reported, an effect mainly attributed to the fluorouracil and folinic acid components of the regimen [32]. Notably, incidents of neutropenic sepsis were rare, indicating that these risks are manageable with appropriate supportive care [32]. Consistent with these observations, Park SJ et al. (2021) documented an increased incidence of neutropenia (58.8% versus 27% in NAPOLI-1), while severe diarrhea was observed less frequently (5.9% versus 13% in NAPOLI-1). This underscores the necessity for proactive management and suggests that treatment modifications may significantly influence the tolerability profile [30]. Moreover, Wainberg ZA et al. (2023) attested that nal-IRI exhibited lower rates of Grade 3–4 peripheral neuropathy and hematological adverse events compared to the nab-PTX plus gemcitabine regimen [28].

Furthermore, emerging evidence suggests that biomarker-driven approaches may enhance the application of nal-IRI in personalized oncology. Glassman DC et al., in 2018, investigated the association between TP53 mutation status and mPFS in patients treated with the nal-IRI plus 5-FU/LV regimen. The study demonstrated that patients harboring wild-type TP53 experienced significantly improved treatment efficacy, with an mPFS of 6.0 months, compared to only 2.2 months in those with TP53 mutations [31]. The underlying biological rationale for these observations likely involves the crucial role of p53 in orchestrating cellular responses to DNA damage. In tumors with an intact *TP53* gene, nal-IRI-induced DNA damage, primarily through the active metabolite of irinotecan, triggers p53 activation, which subsequently upregulates downstream effectors, such as p21^WAF1/CIP1^ [48]. This cascade enforces a robust cell-cycle arrest, ultimately leading to apoptosis when the damage is irreparable. Conversely, in tumors harboring TP53 mutations, the disruption of this critical checkpoint mechanism results in an attenuated p53 response, permitting continued cell-cycle progression, and thereby diminishing the cytotoxic efficacy of nal-IRI [48]. Although definitive conclusions cannot be drawn from studies with limited sample sizes, this preliminary evidence highlights the potential of TP53 status as a predictive biomarker and underscores the critical need for further genomic profiling in larger, controlled patient cohorts to optimize patient stratification and to enhance therapeutic outcomes [31,48]. Similarly, Wainberg ZA et al., in 2023, recommended that future research should seek insight into integrating genomic biomarkers into treatment algorithms to ensure that nal-IRI is administered to patients most likely to derive clinical benefit [28].

### 4.3. Other Nanoparticle-Based Targeted Therapies

The advancement of NP-based targeted therapies has introduced innovative strategies to enhance chemotherapy delivery while diminishing systemic toxicity. In a phase I/II trial, Stathopoulos GP et al. (2006) demonstrated that a nanoliposomal formulation of cisplatin, known as lipoplatin, when combined with gemcitabine, is well tolerated in advanced pretreated PC patients. The study reported promising efficacy, with symptom relief observed in one-third and disease stability achieved in more than half of patients [47]. Moreover, incorporating lipoplatin into the gemcitabine regimen was shown to minimize nephrotoxicity and exhibit manageable myelotoxicity, all while maintaining robust antitumor efficacy [43].

Additionally, Libutti SK et al. (2010) introduced a novel PEGylated colloidal gold NP, CYT-6091, engineered by concurrently conjugating recombinant human tumor necrosis factor alpha (rhTNF-a) and thiolated polyethylene glycol to the surface of 27 nm colloidal gold particles [41]. Evaluated in a phase I dose-escalation clinical trial involving advanced PC patients, CYT-6091 demonstrated selective accumulation in tumor tissues via the enhanced permeability and retention effect. Electron microscopy confirmed intratumoral NP accumulation, thereby supporting its potential role in vascular disruption therapy. The preliminary clinical findings, which revealed partial responses in metastatic ocular melanoma, further underscore the need for additional trials to assess the integration of CYT-6091 with standard chemotherapy protocols [41].

### 4.4. Limitations, Challenges, and Prospective Views

NP-based therapies have emerged as promising candidates for the management of PC; however, several limitations and challenges must be acknowledged when considering their clinical application in real-world patients. A significant concern is biocompatibility, as the materials used in NP formulations may elicit unexpected toxicities or compromise cellular integrity, necessitating a delicate balance between therapeutic efficacy and safety. Additionally, the intricate interactions between NPs and the immune system raise the potential for immunogenic responses that could undermine treatment outcomes and adversely affect patient tolerance. Tumor heterogeneity further complicates the therapeutic landscape, as variations in tumor microenvironment, genetic profile, and phenotypic expression can significantly impact NPs’ delivery, penetration, and overall efficacy. Finally, clinical translation is hampered by regulatory complexities, scalability challenges in manufacturing processes, and the absence of standardized evaluation protocols, all of which underscore the need for rigorous, multidisciplinary research to bridge the gap between preclinical promise and effective clinical implementation.

This scoping review synthesizes the existing literature on NP-based therapeutics for PC; however, several limitations compromise the interpretability and generalizability of its conclusions. One major limitation is the absence of rigorous statistical analyses that would facilitate the evaluation of significant differences among the various NP therapies. This gap limits the ability to discern whether the observed therapeutic effects are truly attributable to the NP interventions or are instead a product of underlying variability. Furthermore, the heterogeneity inherent in clinical trial populations represents a critical challenge. Variations in baseline performance status, differences in previous lines of therapy, and other patient-specific factors can markedly influence treatment outcomes. For instance, patients with a higher baseline performance status or fewer prior treatments might respond differently to NP-based therapies compared to those with more advanced disease or multiple prior therapies. Such discrepancies could raise concerns about the applicability of these findings to real-world clinical settings, where patient populations are diverse. Another concern is the lack of a systematic quality assessment within the review. Without a robust methodological appraisal of the included studies, potential biases stemming from variations in study design, data collection, and outcome reporting may remain unaddressed, further complicating the interpretation of the aggregated data. To address these limitations, we propose a prospective systematic review complemented by a meta-analysis that specifically incorporates subgroup analyses based on patient-level characteristics, such as performance status and treatment history. By employing advanced statistical methods, this approach will enable us to unravel the complex interplay between patient heterogeneity and treatment efficacy. Ultimately, this strategy aims to yield a more nuanced and clinically relevant understanding of how NP therapies impact quality of care and health outcomes in PC, thereby informing clinical decision-making and guiding future research directions.

## 5. Conclusions

Nanoparticle-based therapeutics has emerged as a transformative paradigm in the management of pancreatic cancer. Evidence from multiple clinical trials demonstrates that formulations such as nanoparticle albumin-bound paclitaxel and nanoliposomal irinotecan markedly enhance intratumoral drug delivery, improve overall and progression-free survival, and reduce systemic toxicity compared with conventional chemotherapy. These innovative agents achieve more effective cytotoxicity through enhanced tumor penetration and modulation of the tumor microenvironment, thereby offering promise in overcoming the inherent challenges posed by a dense, fibrotic stroma that traditionally impedes drug efficacy. Furthermore, modifications in dosing strategies have rendered these therapies feasible across diverse patient populations, including elderly patients and those with compromised performance status.

Despite these promising advances, several challenges remain. Unresolved questions include optimizing dosing regimens, identifying and validating predictive biomarkers (such as TP53 status), and ensuring long-term biocompatibility and safety. Moreover, issues related to regulatory approval pathways and manufacturing scalability must be rigorously addressed to bridge the gap between preclinical promise and real-world clinical application.

Over the next decade, nanoparticle-based therapies are expected to become integral to the standard of care for pancreatic cancer. These modalities are poised to serve as critical components in multimodal treatment strategies, working synergistically with chemotherapy, immunotherapy, and targeted agents to reshape therapeutic approaches. Future research must prioritize large-scale, rigorously designed clinical trials and comprehensive meta-analyses that incorporate patient-level subgroup analyses. Such efforts are essential not only for optimizing dosing and sequencing strategies but also for establishing precise patient selection criteria to maximize clinical benefit and improve survival outcomes. This integrative approach heralds a transformative era in personalized oncology, where the strategic deployment of nanoparticle platforms could ultimately redefine the prognosis of one of the most challenging malignancies.

## Figures and Tables

**Table 1:** Population, Intervention, Comparator, Outcome, Time and Study design (PICOTS) table.

P (Population)	Adult (≥18 years) patients with pancreatic cancer
I (Intervention)	Nanoparticle-based pharmacotherapy
C (Comparator)	Conventional pharmacological regimens
O (Outcome)	OS, PFS, DCR, PR, CR, SD, and TEAEs
T (Time)	20 Years (2005–2025)
S (Study design)	Clinical trials, cohorts (retrospective or prospective), case reports

**Table 2:** Comparative summary table of clinical trials and retrospective studies. N: number of participants; NPs: nanoparticles; mOS: median overall survival; mo: months; CI: confidence interval; mPFS: median progression-free survival; DCR: disease control rate; PR: partial remission; CR: complete remission; SD: stable disease; TEAEs: treatment-emergent adverse events; nab-PTX: nanoparticle albumin-bound paclitaxel; nal-IRI: nanoliposomal irinotecan: mPDAC: metastatic pancreatic ductal adenocarcinoma; 5-FU: 5-fluorouracile; LV: leucovorin; NALIRIFOX: nal-IRI, oxaliplatin, 5-FU, LV; FOLFIRINOX: irinotecan, oxaliplatin, 5-FU, LV; mFOLFIRINOX: modified FOLFIRINOX; ECOG PS: Eastern Cooperative Oncology Group Performance Status.

Author, Year	Type of Study	N	Patients	NPs	Regimens	Age (Years)	mOS (mo; 95% CI)	mPFS (mo; 95% CI)	DCR (%, 95% CI)	PR (%, 95% CI)	CR (%, 95% CI)	SD (%, 95% CI)	TEAEs	Conclusions
**Nanoliposomal Irinotecan and Nanoparticle Albumin-Bound Paclitaxel**														
Wainberg ZA et al., 2023 [28]	Phase III randomized clinical trial (NAPOLI 3; NCT04083235)	770	Previously untreated mPDAC	Nanoliposomal irinotecan and nanoparticle albumin-bound paclitaxel	NALIRIFOX (N = 383) vs. nab-PTX + gemcitabine (N = 387)	64 (20–85) vs. 65 (36–82)	11.1 (10.0–12.1) vs. 9.2 (8.3–10.6)	7.4 (6.0–7.7) vs. 5.6 (5.3–5.8)	41.8 (36.8–46.9) vs. 36.2 (21.4–41.2)	42 vs. 36	<1 vs. <1	26 vs. 26	Grade 3–4: neutropenia; diarrhea; hypokalemia in the NALIRIFOX group Grade 3–4: neutropenia; anemia; peripheral neuropathy in the nab-PTX + gemcitabine group	NALIRIFOX promising treatment for mPDAC; improved survival and tolerability; fewer hematological TEAEs
**Nanoliposomal Irinotecan**														
Bockorny B et al., 2021 [29]	Phase IIa clinical trial (COMBAT/KEYNOTE-202; NCT02826486)	43	De novo mPDAC with documented radiographic progression after treatment with first-line gemcitabine-based chemotherapy	Nanoliposomal irinotecan	Motixafortide + pembrolizumab + nal-IRI + 5-FU + LV	68 (40–85)	6.6 (4.5–8.7)	3.8 (1.6–5.1)	63.2 (47.8–78.5)	N/A	N/A	42.1 (26.4–57.8)	Grade 3–4: nausea and vomiting (18.6%); asthenia (16.3%); diarrhea (14%); serious neutropenia (7%); febrile neutropenia (2.3%); reactions at the injection site (4.7%)	Safe; well tolerated; promising efficacy; lower-than-expected rates of neutropenia and infections
Park SJ et al., 2021 [30]	Retrospective cohort study	51	mPDAC previously treated with gemcitabine-based therapy	Nanoliposomal irinotecan	nal-IRI + 5-FU + LV	67 (50–78)	7.0 (6.0–7.9)	2.8 (1.8–3.7)	60.8	5.9	0	54.9	Anemia (84.3%); neutropenia (84.3%; Grade 3–4: 58.8%); nausea (43.1%); diarrhea (23.5%); fatigue (21.6%); febrile neutropenia (7.8%)	Clinical benefits; gemcitabine plus nab-PTX remains a viable first-line treatment option for a significant portion of patients with mPDAC
Glassman DC et al., 2018 [31]	Retrospective study	56	mPDAC previously treated with gemcitabine-based therapy	Nanoliposomal irinotecan	FOLFIRINOX or FOLFOX, followed by nab-PTX + gemcitabine and nal-IRI + 5-FU + LV (Sequence 1) vs. gemcitabine alone or nab-PTX + gemcitabine followed by nal-IRI + 5-FU + LV (Sequence 2)	68 (42–88)	4.1 vs. 9.0	2.2 vs. 4.8	N/A	5	0	41	GI toxicities (nausea and vomiting; diarrhea); fatigue; anorexia; neutropenia; anemia (Grade 3/4)	Confirmed safety and efficacy of nal-IRI + 5-FU/LV for advanced PDAC after gemcitabine-based therapy; earlier use in treatment and absence of irinotecan-refractory disease correlated with improved progression-free survival; dose reductions did not compromise outcomes; genetic predictors of response require further validation; promising OS with sequential integration into combination chemotherapy
Wang-Gillam A et al., 2016 [32]	Phase III randomized clinical trial (NAPOLI-1; NCT01494506)	417	mPDAC previously treated with gemcitabine-based therapy	Nanoliposomal irinotecan	nal-IRI + 5-FU + LV (N = 117) vs. 5-FU + LV (N = 149) vs. nal-IRI monotherapy (N = 151)	63 (57–70) vs. 65 (58–70) vs. 63 (55–69)	6.1 (4.8–8.9) vs. 4.2 (3.3–5.3) vs. 4.9 (4.2–5.6)	3.1 (2.7–4.2) vs. 1.5 (1.4–1.8) vs. 2.7 (2.1–2.9)	N/A	N/A	N/A	N/A	Grade 3–4 neutropenic sepsis and febrile neutropenia (3% vs. 0 vs. 4%); Grade 4 (10% vs. 7% vs. 16%); resulting in death: gastrointestinal toxicity, infectious enterocolitis, septic shock, and disseminated intravascular coagulation with pulmonary embolism	Improves survival and other key efficacy measures in metastatic PC patients previously treated with gemcitabine-based therapy; manageable and mostly reversible safety profile; new treatment option, although its applicability to patients with low performance status remains uncertain
**Nanoparticle albumin-bound paclitaxel**														
Ceelen W et al., 2022 [33]	Phase I clinical trial (NCT03304210)	20	Peritoneal metastases from ovarian, breast, gastric, hepatobiliary, or pancreatic origin	Nanoparticle albumin-bound paclitaxel	Pressurized intraperitoneal aerosol chemotherapy (PIPAC) nab-PTX	57 (49–65)	N/A	N/A	N/A	N/A	N/A	N/A	Hematological toxicity (moderate); Grade 3 neutropenia	Safety and potential effectiveness for advanced, unresectable peritoneal metastases; well-tolerated dosing; stable patient quality of life; promising anticancer activity
Sohal DPS et al., 2021 [34]	Phase II randomized clinical trial (NCT02562716)	102	Treatment-naïve PDAC with no metastases	Nanoparticle albumin-bound paclitaxel	mFOLFIRINOX (N = 55) vs. nab-PTX + gemcitabine (N = 47)	66 (44–76) vs. 64 (46–75)	23.2 (17.6–45.9) vs. 23.6 (17.8–31.7)	N/A	N/A	N/A	N/A	N/A	Neoadjuvant: neutropenia (19% vs 27%); (11% vs 4%)	Safety and efficacy; no improved OS compared to historical adjuvant trials
Azmi AS et al., 2020 [35]	Phase Ib study (NCT02178436)	5	mPDAC not treated with chemotherapy	Nanoparticle albumin-bound paclitaxel	Selinexor + gemcitabine + nab-PTX	N/A	N/A	N/A	N/A	N/A	N/A	N/A	N/A	Synergy between selinexor and GEM-nab-PTX in PDAC models, including stem cells and patient-derived xenografts
Hasegawa R et al., 2019 [36]	Clinical trial (UMIN000018907)	27	Unresectable advanced PC + age ≥ 75 years	Nanoparticle albumin-bound paclitaxel	nab-PTX + gemcitabine	77 (75–85)	10.3 (8.2–12.5)	7.0 (6.0–8.1)	N/A	44.4	N/A	48.1	Grade 3–4: hemotoxic (51.9%); non-hemotoxic (59.3%): peripheral nerve disorder (22.2%)	Improved progression-free survival in elderly patients; favorable disease control even in stage IV cases; feasible with proper dose management; elderly patients more susceptible to non-hemotoxic TEAEs
Macarulla T et al., 2019 [37]	Phase I trial	24	Locally advanced or advanced PDAC; ECOG PS of 2	Nanoparticle albumin-bound paclitaxel	nab-PTX at 150 mg/m^2^ (arm A) or 125 mg/m^2^ (arm C) + gemcitabine; nab-PTX at 100 mg/m^2^ (arm B) or 125 mg/m^2^ (arm D) + gemcitabine	N/A	N/A	N/A	N/A	N/A	N/A	N/A	N/A	Not designed for direct comparison; both arms showed similar efficacy and toxicity; potential treatment option in clinical practice
	Phase II randomized trial (NCT02382263)	221	Locally advanced or advanced PDAC; ECOG PS of 2		nab-PTX at 100 mg/m^2^ (arm B; N = 111) or 125 mg/m^2^ (arm D; N = 110) + gemcitabine	71 (43–89) vs. 68 (35–84)	7.7 (6.3–9.1) vs. 9.8 (7.5–11.8)	5.4 (4–6.9) vs. 6.6 (5.6–7.6)	64.9 (56–73.7) vs. 71.8 (63.4–80.2)	20.7 vs. 21.8	0 vs. 0.9	N/A	Hematological toxicity (neutropenia); fatigue; peripheral neuropathy	
Von Hoff DD et al., 2013 [38]	Phase III Randomized control trial (MPACT; NCT00844649)	861	mPDAC not treated with chemotherapy	Nanoparticle albumin-bound paclitaxel	nab-PTX + gemcitabine (N = 431) vs. gemcitabine monotherapy (N = 430)	63 (27–88)	8.5 (7.89–9.53) vs. 6.7 (6.01–7.23)	5.5 (4.5–5.9) vs. 3.7 (3.6–4.0)	48 (43–53) vs. 33 (28–37)	23 vs. 7	<1 vs. 0	20 vs. 26	Grade 3–4: neutropenia; leukopenia; fatigue; peripheral neuropathy (mostly in nab-PTX cohort)	Significantly improved survival; benefits observed across multiple time points and subgroups; increased myelosuppression and peripheral neuropathy, reversible; potential as an effective treatment option
Hosein PJ et al., 2013 [39]	Phase II clinical trial (NCT00691054)	19	Advanced PC that progressed on gemcitabine-based therapy with unresectable locally advanced or metastatic disease	Nanoparticle albumin-bound paclitaxel	nab-PTX monotherapy	61 (24–80)	7.3 (2.8–15.8)	1.7 (1.5–3.5)	5	5	0	32	Grades 3–4 neutropenia (26%); Grades 3–4 anemia (11%); neutropenic fever (11%); hypocalcemia	The nab-PTX monotherapy demonstrated preliminary activity in a subset of patients and was well tolerated
Von Hoff DD et al., 2011 [40]	Phase I/II clinical trial (NCT00398086.)	67	Previously untreated advanced PC	Nanoparticle albumin-bound paclitaxel	100 (N = 20), 125 (N = 44; Results), or 150 (N = 3) mg/m^2^ nab-PTX + gemcitabine	62 (30–86) vs. 61 (28–78) vs. 69 (53–72)	12.2 (8.9–17.9)	7.9 (5.8–11.0)	68	48	0	20	Grade 3–4 fatigue (21%); sensory neuropathy (15%); neutropenia (67%); leukopenia (44%); thrombocytopenia (23%)	Favorable safety and encouraging antitumor activity; patient selection may influence outcomes
**Other Nanoparticle-Based Targeted Therapies**														
Libutti SK et al., 2010 [41]	Phase I clinical trial	3	Advanced-stage PC patients	CYT-6091	PEGylated colloidal gold nanoparticle carrying rhTNF-a	N/A	N/A	N/A	N/A	N/A	N/A	N/A	Grade 3–4 lymphopenia (89%); hypoalbuminemia (17%); hypokalemia (17%); hypophosphatemia (17%); hyperbilirubinemia (17%), increased AST (17%)	Promising tumor targeting; potential benefits when administered systemically before chemotherapy or surgery, particularly for solid tumors
Hamaguchi T et al., 2007 [42]	Phase I clinical trial	11	PC refractory to conventional chemotherapy	NK105	Polymeric micellar nanoparticle paclitaxel	57 (43–72)	N/A	N/A	N/A	N/A	N/A	N/A	Grade 3–4: neutropenia; Grade 1–2: fever; nausea; fatigue; stomatitis; rash; alopecia	Reduction in the size of metastatic lesions; minimal severity of adverse events; favorable therapeutic response and manageable safety profile
Stathopoulos GP et al., 2006 [43]	Phase I/II clinical trial	24	Advanced PDAC after chemotherapy pretreatment and recurrent or non-responsive disease	Nanoliposomal cisplatin	Lipoplatin + gemcitabine	66 (47–80)	4 (2–8)	N/A	N/A	8.3	N/A	58.3	Grade 3 myelotoxicity (50%)	Well tolerated in advanced pretreated PC patients; promising efficacy with symptom relief and disease stability

**Table 3:** Comparative summary of case reports. NPs: nanoparticles; nab-PTX: nanoparticle albumin-bound paclitaxel; nal-IRI: nanoliposomal irinotecan; SCC: squamous-cell carcinoma; mPDAC: metastatic pancreatic ductal adenocarcinoma; 5-FU: 5-fluorouracile; LV: leucovorin.

Author, Year	Type	Disease	Gender	Age	NPs	Regimen	Conclusions
Huang X et al., 2021 [44]	Case report	Stage IV primary SCC of the pancreas harboring a deleterious BRCA2 somatic mutation	F	52	NP albumin-bound paclitaxel	nab-PTX + cisplatin	Significant tumor reduction; prolonged survival; improved tumor resectability; the longest reported survival for metastatic pancreatic SCC to date
Otsubo M et al., 2021 [45]	Case report	Stage IV PC with hepatic and lymph node metastases	M	70	NP albumin-bound paclitaxel	nab-PTX + gemcitabine	Taxane-related cystoid macular edema (CME) as a rare but notable side effect of nab-PTX, affecting bilateral central vision; CME resolved after stopping nab-PTX, with no intervention other than topical dorzolamide
Assi HA et al., 2020 [46]	Case report	T4, N1, and M1 primary pancreatic adenocarcinoma	F	57	Nanoliposomal irinotecan and NP albumin-bound paclitaxel	nab-PTX + gemcitabine followed by nal-IRI + 5-FU + LV	Rare instance of long-term survival in an mPDAC patient treated with nal-IRI + 5-FU/LV; despite initial disease progression, sustained response for over two years, receiving 58 cycles without dose adjustments; PFS and OS reached 31 and 40 months, respectively; potential clinical benefits

## Supplementary Materials

The following supporting information can be downloaded at https://www.mdpi.com/article/10.3390/cancers17101726/s1, Table S1: Preferred Reporting Items for Systematic reviews and Meta-Analyses Extension for Scoping Reviews (PRISMA-ScR) checklist.
